# Retraction: How organizations create employee based brand equity: mediating effects of employee empowerment

**DOI:** 10.3389/fpsyg.2025.1675464

**Published:** 2025-08-07

**Authors:** Frontiers Editorial Office

The journal retracts the 2022 article cited above.

Frontiers Research Integrity Auditing team has investigated and uncovered a network of authors and editors who conducted peer review with undisclosed conflicts of interest and who have engaged in citation manipulation. The investigation identified this article as one for which the integrity of the peer review process has been undermined, resulting in the loss of confidence in the article's findings.

The authors received a communication regarding the retraction and were given a chance to respond, with some discussions still ongoing. This exchange has been recorded by the publisher. The investigation was not able to determine whether all authors, editors, or reviewers were aware of or involved in the misconduct, but this misconduct was significant enough to determine that the scientific integrity of the article cannot be guaranteed.

In adherence to the recommendations of the Committee on Publication Ethics (COPE), the article is retracted. The retraction was approved by the Chief Executive Editor at Frontiers and the Field Chief Editor of Frontiers in Psychology.

